# Selected Thieno[2,3-*d*] Pyrimidine Derivatives Target Breast Cancer Cell Proliferation and Membrane Organization

**DOI:** 10.3390/molecules31142435

**Published:** 2026-07-11

**Authors:** Aleksandrina Nesheva, Ivan Iliev, Anelia Mavrova, Denitsa Yancheva, Aneliya Kostadinova, Severina Semkova, Albena Momchilova, Iana Tsoneva, Biliana Nikolova, Galya Staneva

**Affiliations:** 1 Institute of Biophysics and Biomedical Engineering, Bulgarian Academy of Sciences, Acad. G. Bonchev Str., Bl. 21, 1113 Sofia, Bulgaria; nescheva@gmail.com (A.N.); aneliakk@yahoo.com (A.K.); seveina.yordanova@gmail.com (S.S.); albena_momchilova@abv.bg (A.M.); itsoneva@bio21.bas.bg (I.T.); 2 Institute of Experimental Morphology, Pathology and Anthropology with Museum, Bulgarian Academy of Sciences, Acad. G. Bonchev Str., Bl. 25, 1113 Sofia, Bulgaria; taparsky@abv.bg; 3 Department of Organic Synthesis, Faculty of Chemical Technologies, University of Chemical Technology and Metallurgy, S8 Kliment Ohridski Blvd., 1756 Sofia, Bulgaria; anmav@abv.bg; 4 Institute of Organic Chemistry with Centre of Phytochemistry, Bulgarian Academy of Sciences, Acad. G. Bonchev Str., Bl. 9, 1113 Sofia, Bulgaria; denitsa.pantaleeva@orgchm.bas.bg

**Keywords:** breast cancer cells, thieno[2,3-*d*]pyrimidine scaffold, selective cytotoxicity, clonogenic survival, cell-cycle arrest, plasma membrane organization, actin cytoskeleton

## Abstract

Breast cancer remains one of the leading causes of cancer-related mortality among women worldwide, highlighting the urgent need for novel therapeutic agents with improved efficacy and selectivity. In this study, we investigated the anticancer potential of selected thieno[2,3-*d*]pyrimidine-based hybrids in breast cancer cell models with different metastatic potential. The compounds were evaluated for their effects on cell viability, clonogenic survival, migration, cell-cycle progression, cytoskeletal organization, and plasma membrane organization. Several derivatives exhibited concentration-dependent antiproliferative activity, inhibited colony formation and cell migration, disrupted cytoskeletal integrity, and modulated plasma membrane organization. Cell-cycle analysis revealed compound- and cell line-dependent alterations in the distribution of cells across the G1, S, and G2/M phases. Among the tested compounds, derivatives 1, 5, and 6 displayed the most favorable biological profiles, combining antiproliferative activity with moderate selectivity toward breast cancer cells, whereas compound **2** exerted the most pronounced effects on plasma membrane organization and cell migration. Collectively, these findings identify selected thieno[2,3-*d*]pyrimidine-based hybrids as promising lead compounds for the development of novel anticancer agents targeting both tumor cell proliferation and plasma membrane organization.

## 1. Introduction

Breast cancer remains one of the leading causes of cancer-related mortality among women worldwide [1]. Despite significant advances in early diagnosis and therapeutic strategies, including surgery, chemotherapy, radiotherapy, and targeted therapies, treatment resistance, systemic toxicity, and tumor recurrence continue to represent major clinical challenges. Therefore, the development of novel anticancer agents with improved selectivity toward tumor cells and reduced side effects remains an important objective in cancer research.

In vitro cell culture models are a fundamental tool for studying the molecular mechanisms underlying breast cancer development and for evaluating the biological activity of novel therapeutic agents. Among the most widely used models are the human breast cancer cell lines MCF-7 and MDA-MB-231, which represent different molecular subtypes and levels of aggressiveness. The MCF-7 cell line is one of the most extensively studied models of breast cancer. These cells are estrogen receptor-positive (ER^+^) and exhibit characteristics typical of luminal breast cancer, including hormone-dependent proliferation and relatively low invasive potential. Because of these properties, MCF-7 cells are often used to study hormone-responsive signaling pathways and to evaluate compounds targeting estrogen-dependent tumor growth [2,3].

In contrast, MDA-MB-231 cells represent a more aggressive triple-negative subtype of breast cancer. These cells lack expression of estrogen receptor (ER), progesterone receptor (PR), and human epidermal growth factor receptor-2 (HER2), which limits the effectiveness of targeted hormone therapies. MDA-MB-231 cells are characterized by a highly invasive phenotype, increased migratory and metastatic potential, making them a valuable model for studying tumor progression and resistance mechanisms. Their aggressive biological behavior and molecular characteristics make this cell line widely used in studies focused on identifying new therapeutic strategies for difficult-to-treat breast cancers [4].

To assess the cytotoxicity, selectivity and safety profile of the newly synthesized compounds, non-tumorigenic cell lines were included in the studies as controls. The MCF-12F cell line is a non-tumorigenic human breast epithelial cell line derived from breast tissue obtained during reduction mammoplasty. These cells exhibit epithelial morphology, express cytokeratin markers typical of breast epithelial cells, and do not form tumors in vivo, making them suitable as a normal cell model for comparison with cancer cell lines [5]. The use of both cancerous and non-cancerous breast cell lines allows for the evaluation of not only the anticancer efficacy of novel compounds but also their selectivity towards malignant cells. Compounds demonstrating selective cytotoxicity towards cancer cells while sparing normal epithelial cells are particularly desirable as they may lead to improved therapeutic outcomes with reduced adverse effects.

Uncontrolled cell proliferation, survival, and migration in cancer—particularly breast cancer—are predominantly governed by dysregulated intracellular signaling pathways mediated by protein kinases, which orchestrate critical cellular processes including growth, differentiation, and apoptosis. Aberrant kinase signaling is a defining hallmark of oncogenesis, driving aggressive tumor progression, metastasis, and therapeutic resistance across multiple cancer types, including HER2-positive and triple-negative breast cancers [6,7,8].

Consequently, tyrosine kinase inhibitors have revolutionized targeted anticancer therapy by precisely blocking kinase catalytic activity, thereby disrupting essential downstream oncogenic signals that sustain tumor development and survival. Compounds bearing the thieno[2,3-*d*]pyrimidine backbone exhibit a diversity of biological activities, such as anticancer, anti-inflammatory, antimicrobial, and antiviral activities [9,10]. The anticancer effect of thienopyrimidines relates to different mechanisms: kinase inhibitory effect, tubulin polymerization inhibition and nonselective antiproliferative activity [11,12,13]. Given the limitations of traditional small-molecule inhibitors, current drug discovery efforts increasingly focus on novel heterocyclic scaffolds capable of modulating these pathways through multi-targeted or alternative binding site interactions [14]. Pyrimidine-based derivatives, particularly fused heterocyclic systems like thienopyrimidines, have emerged as prominent scaffolds in medicinal chemistry due to their structural similarity to purine-based kinase inhibitors and their ability to interact with kinase catalytic domains [15,16,17]. These structural analogs of naturally occurring purines leverage their unique electronic properties to disrupt essential signaling cascades [18] and demonstrate promising anticancer activity—especially against breast cancer cell lines such as MCF-7—through inhibition of cell proliferation, induction of apoptosis, cell-cycle arrest, and suppression of oncogenic signaling pathways [14,16]. 

**Scheme 1:**
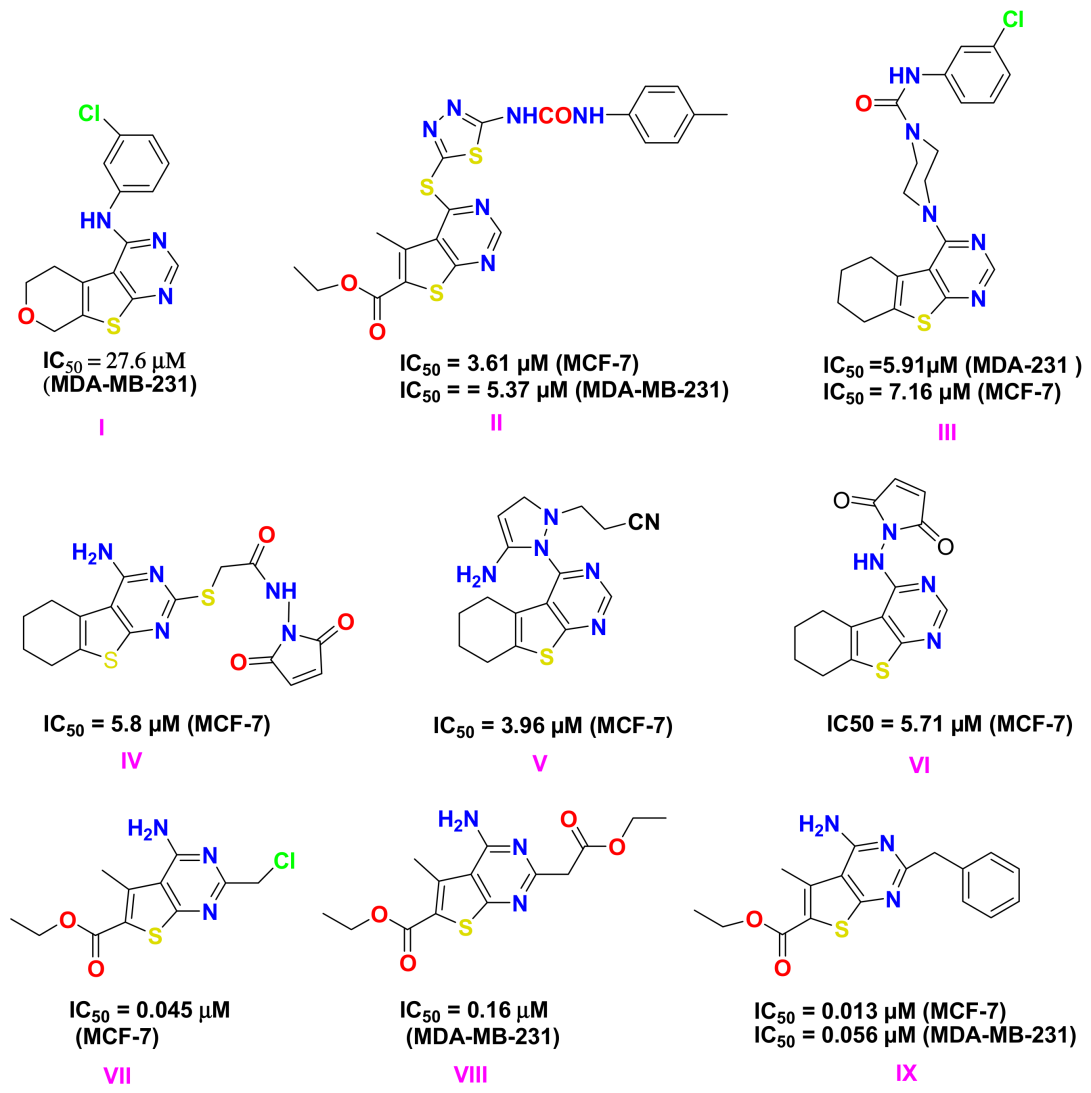
Thieno[2,3-*d*]pyrimidines demonstrating MCF-7 and MDA-MB-31 cell lines anti-cancer activity.

A series of novel thieno[2,3-*d*]pyrimidine derivatives were synthesized and investigated for their cytotoxicity. Compound **I** (Scheme 1) showed the strongest cytotoxic activity against MDA-MB-231 cell line (IC_50_ = 27.6 µM) [19]. Thieno[2,3-*d*]pyrimidine **II** containing substituted thiadiazole fragments at the 4th position was reported to possess strong cytotoxicity against MCF-7 breast cancer cells (IC_50_ = 3.61 µM) and the MDA-MB-231 cells (IC_50_ = 5.37 µM) [12]. Derivative **III** in which the amino group at the 4th position is included in a piperazine ring have exhibited cytotoxicity against MCF-7 and MDA-MB-231, and demonstrate significant inhibitory activity against VEGFR-2, similar to that of sorafenib (IC_50_: 53.63 ± 3.14 nM) [20]. Through the MTT assay of novel 4-aminothieno[2,3-*d*]pyrimidines and triazolothienopyrimidines, compound **IV** has been identified as the most active against MCF-7 [21]. Compounds **V** and **VI** revealed anticancer and topoisomerase-II inhibitory activities [22]. The present research work is a continuation of our previous studies concerning the anti-proliferative and apoptotic effects of 4-amino-thienopyrimidines against MCF-7 and MDA-MB-31 cell lines. The most active of these compounds have IC_50_ = 0.045 and 0.11 μM, respectively [23,24] (Scheme 1).

These molecules act as effective competitive inhibitors within the ATP-binding pockets of critical oncogenic kinases, including EGFR, HER2, VEGFR-2, and Aurora B [12,20,25]. Suppression of these signaling axes effectively halts cell proliferation, triggers cell cycle arrest at the G1 or G2/M phases, and induces caspase-dependent apoptosis or alternative, non-apoptotic cell death pathways mediated by severe mitochondrial stress [26]. Structural modifications of the pyrimidine core significantly influence biological activity, enabling the design of compounds with improved potency, selectivity, and favorable pharmacological properties [27]. In addition to their intracellular targets, the fused heteroaromatic scaffold and hydrophobic substituents of thieno[2,3-*d*]pyrimidines may also facilitate interactions with biological membranes. Such membrane partitioning has been reported for several small-molecule anticancer agents and may influence membrane lipid organization, receptor clustering, and membrane-associated signaling independently of direct kinase inhibition [28,29,30]. These observations raise the possibility that membrane-associated effects contribute to the overall anticancer activity of thieno[2,3-*d*]pyrimidines. However, this aspect has received considerably less attention than their well-established intracellular mechanisms of action. The plasma membrane is not merely a passive structural barrier but a dynamic platform that regulates receptor signaling, membrane protein function, cytoskeletal organization, and cell migration. Alterations in membrane organization have been increasingly recognized as important contributors to cancer progression, metastatic behavior, and therapeutic response [15,16,17]. Beyond direct kinase inhibition, these agents modulate the microenvironment by disrupting protein-protein interactions within scaffold-mediated signaling complexes, thereby preventing the recruitment of essential downstream effectors [31,32,33,34,35]. Consequently, investigating membrane remodeling alongside conventional biological endpoints may provide complementary mechanistic insight into the anticancer activity of novel thienopyrimidine derivatives beyond their established intracellular targets.

Although several thieno[2,3-*d*]pyrimidine derivatives have previously been evaluated in other cancer models, including oral carcinoma, their biological activity has not been comprehensively investigated in breast cancer models representing different molecular subtypes and metastatic potential. Moreover, little is known about their effects on plasma membrane organization and cytoskeletal architecture, two closely interconnected determinants of cell signaling, migration, and metastatic behavior. Therefore, integrating the assessment of antiproliferative activity with analyses of plasma membrane organization, cell migration, clonogenic survival, cell-cycle progression, and cytoskeletal organization may provide novel mechanistic insights into the biological activity of these compounds in breast cancer. These considerations provide the rationale for the present study. Based on these considerations, the present study was designed to investigate the biological activity of selected thieno[2,3-*d*]pyrimidine derivatives in the luminal breast cancer cell line MCF-7 and the highly metastatic triple-negative breast cancer cell line MDA-MB-231. In addition to evaluating their antiproliferative activity, we examined their effects on plasma membrane organization, migration, clonogenic survival, cell-cycle progression, and cytoskeletal organization to explore whether membrane remodeling contributes to their overall anticancer activity. The inclusion of the non-tumorigenic mammary epithelial cell line MCF-12F further enabled the assessment of tumor selectivity and potential therapeutic relevance. 

By integrating conventional biological assays with the assessment of plasma membrane organization, the present study aims to provide a more comprehensive evaluation of the mechanisms underlying the anticancer activity of thieno[2,3-*d*]pyrimidine derivatives and to determine whether membrane remodeling contributes to their biological effects.

## 2. Results

### 2.1. Antiproliferative Effects of the Tested Thienopyrimidine Derivatives 

**Figure 1:**
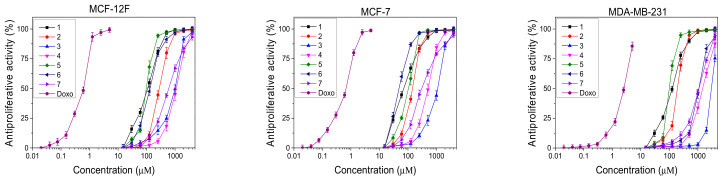
Antiproliferative effects of the tested compounds. Dose–response curves representing the effects of the tested compounds on the viability of normal mammary epithelial cells (MCF-12F), luminal A breast cancer cells (MCF-7), and basal B breast cancer cells (MDA-MB-231) after 72 h of exposure. Cell viability was assessed using the MTT assay (*n* = 6). The error bars represent standard error of the mean (SEM). IC_50_ values were calculated from the nonlinear regression of cell viability versus concentration. Detailed statistical analyses are provided in the Supplementary Materials.

Compounds (**1**−**7**) were tested for their antiproliferative effect on a panel of breast cell lines, as described above. In this study Doxorubicin (Doxo) was used as a positive control, because it is a clinically established chemotherapeutic agent for breast cancer and serves as a standard reference compound for evaluating the anticancer activity of novel molecules in in vitro studies. The concentration of tested compounds was in wide range. The cells were incubated with the corresponding compounds for 24 h at 37 °C, 5% CO_2_ and 95% humidity. The results are presented in Figure 1.

The strongest antiproliferative effect of the pyrimidine-derived compounds was observed in breast cancer cell lines MCF-7 (luminal A subtype) followed by MDA-MB-231 (basal B subtype), and in the non-tumorigenic mammary epithelial cell line MCF-12F. 

Treatment of breast cancer cells with pyrimidine derivatives produced a clear dose-dependent reduction in cell viability. Among the evaluated derivatives, compound **6** exerted the most pronounced antiproliferative effect on MCF-7 cells, with an IC_50_ of approximately 49 μM, followed by compounds **1**, **5** and **2** which displayed intermediate IC_50_ values, ranging from 71.56 to 144.3 μM. As expected, the highly aggressive cell line MDA-MB-231 showed reduced sensitivity to the tested compounds. Most active was compound 5 showing IC_50_ value of 99.29 μM, followed by compound **1** with IC_50_ value of 125.62 μM. Although cell viability decreased with increasing concentration, the inhibitory effects were generally less pronounced compared with MCF-7 cells. This observation is consistent with the known drug resistance and invasive phenotype of triple-negative breast cancer cells. Compounds **3** and **4** showed IC_50_ values higher than 1 mm in MCF-12F, indicating minimal cytotoxicity toward normal mammary cells at pharmacologically relevant concentrations. As expected, the reference drug Doxorubicin displayed substantially higher potency, producing a marked decrease in cell viability at considerably lower concentrations. However, unlike the tested pyrimidine derivatives, Doxorubicin affected both normal and cancer cells with similar efficiency, highlighting the limited selectivity of conventional chemotherapeutic agents.

Importantly, the non-tumorigenic MCF-12F cells exhibited significantly higher survival rates under identical experimental conditions. In most cases, cell viability remained above 70–80%, even at concentrations that markedly reduced cancer cell viability. This differential response suggests that the tested compounds possess a degree of selectivity toward malignant cells, an essential property for the development of safer anticancer agents.

To further assess tumor selectivity, the selectivity index (SI) was calculated as the ratio between IC_50_ values in normal cells and tumor cells, shown in Table 1. Higher SI values indicate stronger selectivity toward cancer cells. Compounds exhibiting a SI greater than 1.0 were considered to have preferential activity against the tested breast cancer cell lines, suggesting a favorable therapeutic window for further investigation [36]. 

The highest selectivity toward MCF-7 cells was observed for compound **6**, with a selectivity index of 2.69, indicating that this compound was nearly three times more toxic to cancer cells than to normal cells. Compounds **4** and **7** also demonstrated notable selectivity, with SI values of 2.14 and 1.99, respectively. In contrast, compounds **3** and **5** showed limited selectivity, with SI values below 1, indicating comparable toxicity toward both cancerous and non-cancerous cells. Selectivity toward MDA-MB-231 cells was generally lower for all compounds. The highest SI value in this model was observed for compound **2** (SI = 1.45), suggesting modest preferential toxicity toward triple-negative breast cancer cells. In comparison, the reference drug Doxorubicin showed relatively low selectivity, with SI values of 0.89 for MCF-7 and 0.24 for MDA-MB-231 cells, indicating strong cytotoxic effects on both normal and cancer cell lines.

Based on the antiproliferative activity and selectivity index (SI) analysis, several representative compounds were selected for further biological investigations. Compounds **2**, **4**, **5**, and **6** were chosen for studies in the MCF-12F and MCF-7 cell lines, as they exhibited distinct biological profiles, including moderate to strong antiproliferative activity and varying degrees of tumor selectivity. In particular, compound **6** showed the highest selectivity toward MCF-7 cells (SI = 2.69), while compounds **2** and **4** demonstrated relevant cytotoxic activity and selectivity toward cancer cells, making them suitable candidates for further mechanistic studies. Compound **5** was additionally included due to its relatively strong antiproliferative activity in both cancer cell lines, allowing comparison with more selective derivatives. For the more aggressive MDA-MB-231 triple-negative breast cancer model, only compounds **2** and **4** were selected, as these derivatives showed the most selectivity in this cell line. This selection strategy allowed the evaluation of compounds with different activity profiles in order to better understand their biological effects on breast cancer cells.

### 2.2. Effects of Tested Thienopyrimidine-Derived Compounds on Clonogenic Survival

**Figure 2:**
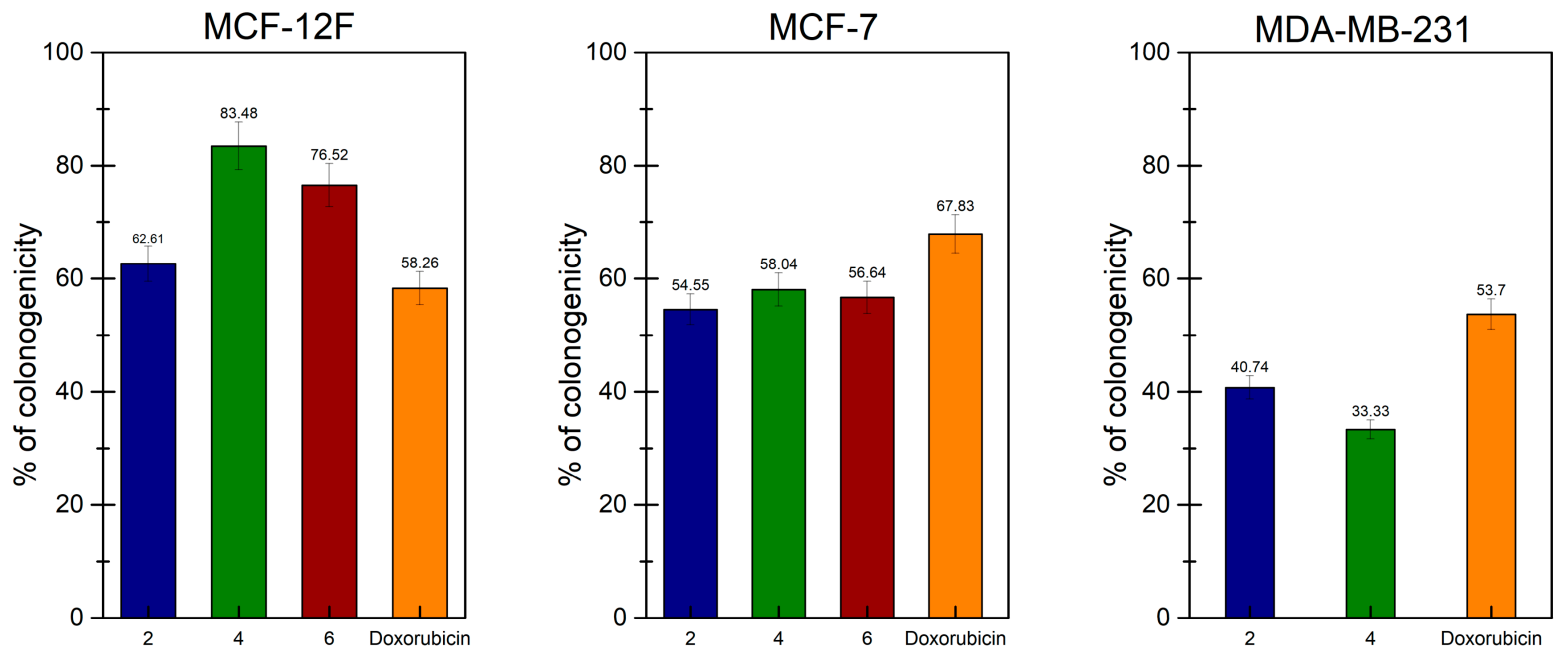
Percentage clonogenicity of breast cell lines after treatment with pyrimidine-derived compounds, calculated from the number of colonies formed after treatment to those formed in untreated control cells (set as 100%).

To evaluate the long-term proliferative capacity and clonogenic survival of cancer cells following treatment, a colony formation assay was performed. This method assesses the ability of single cells to retain their dividing potential and form colonies after prolonged incubation, providing insight into the long-term cytotoxic or cytostatic effects of anticancer agents. Data are presented in Figure 2.

Treatment with the selected pyrimidine-derived compounds resulted in a decrease in clonogenic survival across all tested cell lines, although the magnitude varied by their type. In non-tumorigenic MCF-12F cells, clonogenicity ranged from approximately 58% to 83%, indicating moderate inhibition of colony-forming capacity; compound **6** produced the strongest effect (~58%, similar to Doxorubicin), while compound **4** was weaker (~83%). In MCF-7 breast cancer cells, all compounds reduced clonogenicity relative to untreated controls, with compound **2** showing the lowest value, followed closely by compounds **6** and **4**. Notably, Doxorubicin yielded ~67% clonogenicity, suggesting a comparatively weaker effect. In high-metastatic cell line MDA-MB-231, compound **4** exerted the strongest inhibition, while compound **2** reduced clonogenicity to ~40%; Doxorubicin achieved a moderate ~53% reduction. Treatment of high-metatstic cell line MDA-MB-231 with IC_50_ dose of compound **4** induced the strongest clonogenic effect among all compounds.

### 2.3. Assessment of the Plasma Membrane Lipid Order

**Figure 3:**
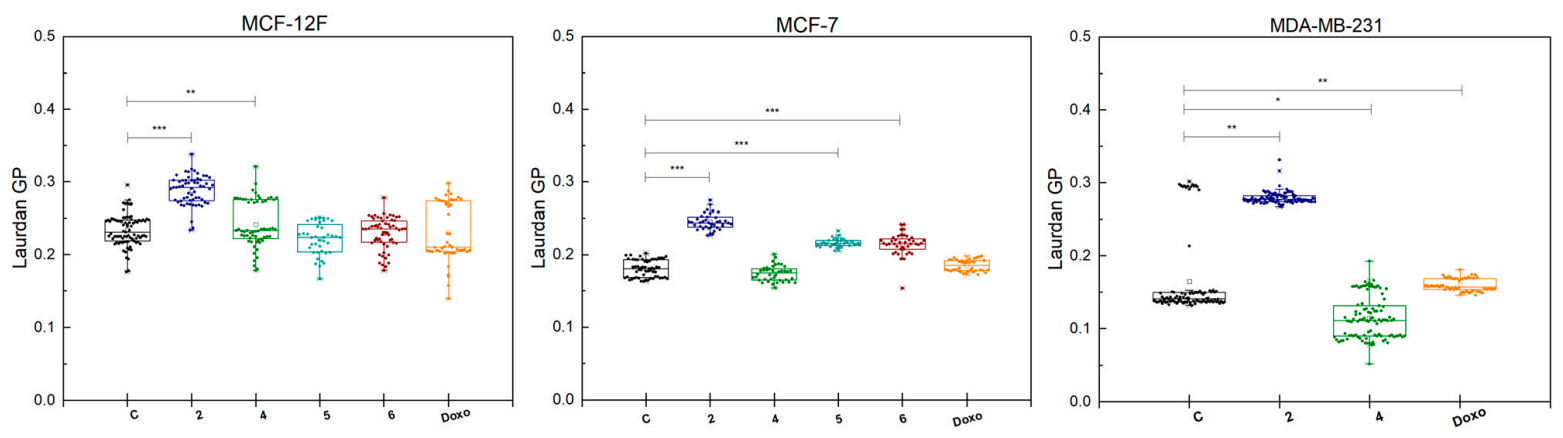
Laurdan GP of untreated and treated with the compounds cell lines. Only statistically significant differences between compound-treated groups and the untreated control are indicated in the graph. A *p*-value < 0.05 was considered statistically significant. Statistical significance is indicated as follows: * *p* < 0.05; ** *p* < 0.01; *** *p* < 0.001.

Changes in plasma membrane lipid organization were evaluated using Laurdan fluorescence spectroscopy, which measures membrane packing through the generalized polarization (GP) parameter. An increase in GP values reflects higher membrane order and reduced lipid fluidity. The obtained results (Figure 3) demonstrated alterations in membrane lipid organization across the investigated cell lines. In MCF-7 and MDA-MB-231 cells, treatment with the pyrimidine-derived compounds resulted in compound-dependent changes in GP values compared with untreated controls, indicating altered membrane order. Compound **2** produced the most pronounced increase in Laurdan GP in both cancer cell lines, suggesting a substantial shift toward more ordered lipid packing within the plasma membrane. Compounds **4** and **6** also modulated GP values, although the magnitude of the effect was smaller compared with compound **2**. In contrast, treatment with the reference drug Doxorubicin resulted in modest changes in GP values that were largely nonselective across the tested cell lines. Notably, the MCF-12F non-tumorigenic epithelial cells showed only minimal changes in GP values following treatment with the tested compounds, indicating that the pyrimidine derivatives have a limited effect on the membrane organization of normal cells. This observation suggests a preferential impact of the compounds on cancer cell membranes, consistent with their selective antiproliferative activity.

### 2.4. Migratory Activity

**Figure 4:**
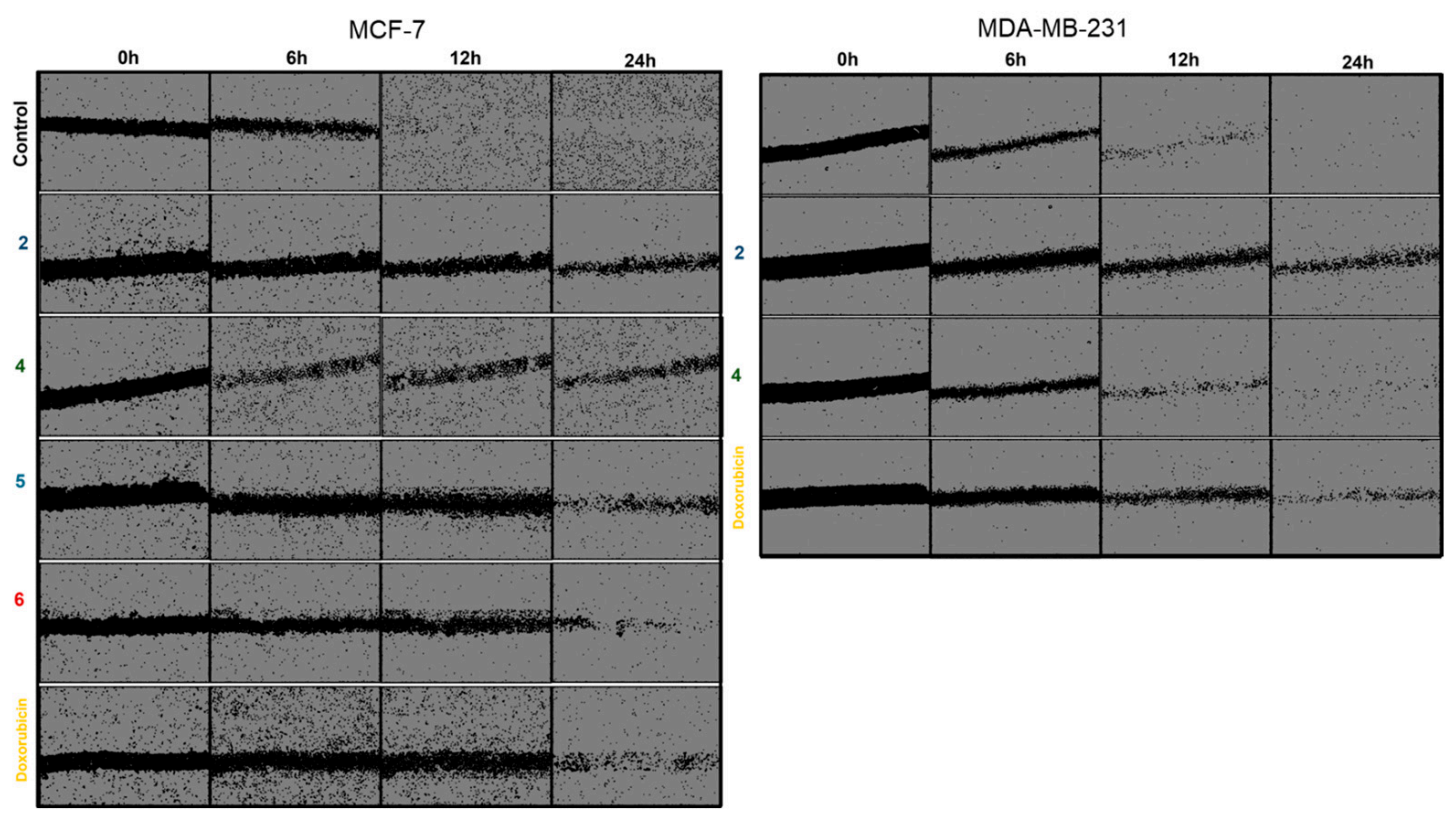
Cell migration assay of the low metastatic cancer cell line MCF-7 and the high metastatic cancer cell line MDA-MB-231 at 0 h, 6 h, 12 h and 24 h with and without pyrimidine-derived compounds and positive control Doxo.

Representative images of the wound-healing assay are shown in Figure 4. In the untreated control groups, both MCF-7 and MDA-MB-231 cells displayed progressive closure of the scratch area over time, confirming active cell migration. As expected, the effect was more pronounced in MDA-MB-231 cells, where the wound area became markedly narrower already after 6 h and was almost completely closed after 24 h, consistent with the highly migratory phenotype of this cell line. In MCF-7 cells, wound closure was also evident but progressed more slowly than in MDA-MB-231 cells. Treatment with the pyrimidine-derived compounds visibly delayed wound closure in both breast cancer cell lines. In MCF-7 cells, compound **2** produced the most apparent inhibitory effect, as the scratch area remained clearly open even after 24 h. Compounds **4**, **5**, and **6** also reduced wound closure relative to the untreated control, although the effect was less pronounced. A similar tendency was observed in MDA-MB-231 cells, where compound **2** again caused the strongest visible inhibition of wound closure, whereas compound **4** showed a weaker but still noticeable effect. Treatment with Doxorubicin also delayed migration compared with the control, although its effect was not consistently stronger than that of the most active pyrimidine derivative.

**Figure 5:**
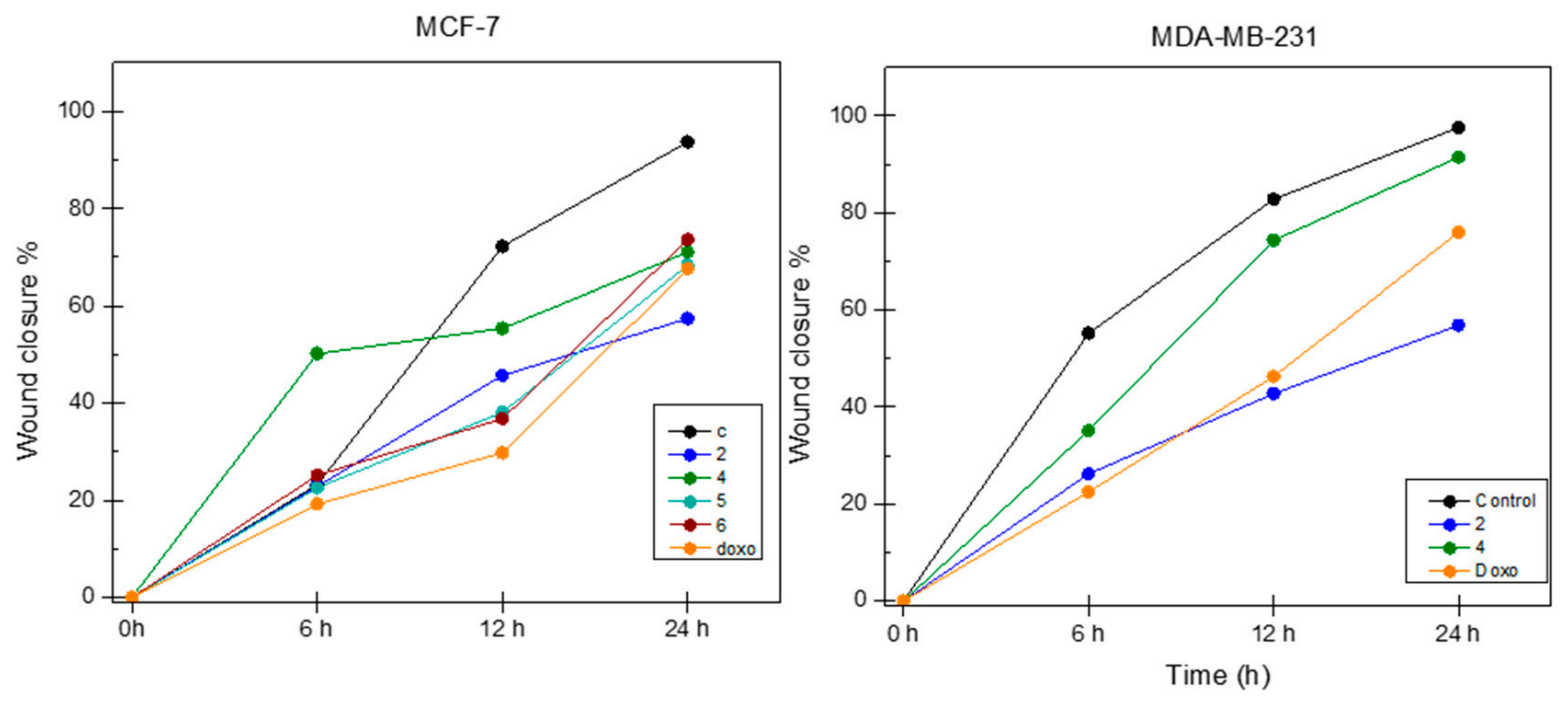
Calculated wound area presenter as a percentage of the initial wound area at 0 h. A statistically significant difference is obtained for all measurements compared to control at both 12 and 24 h (*p* < 0.001).

Quantitative analysis of wound closure is presented in Figure 5. The slope of each curve reflects the rate of wound closure, thus showing the migration speed of the cells over time. A steeper slope indicates faster migration, whereas a flatter slope indicates slower wound closure and reduced migratory activity.

### 2.5. Cell-Cycle Analysis

**Figure 6:**
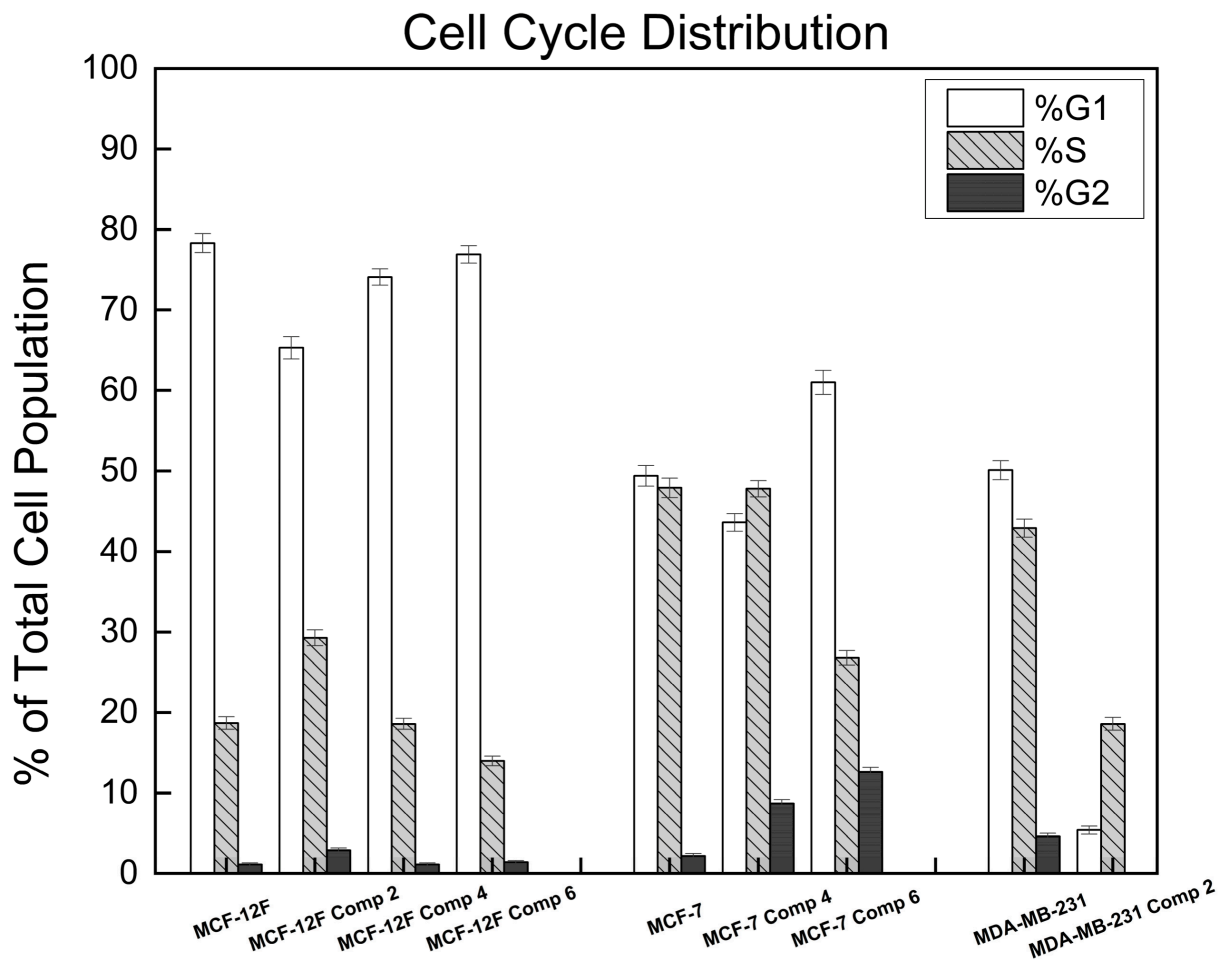
Cell cycle distribution following treatment with selected compounds.

The cell-cycle analysis (Figure 6) demonstrates that the tested compounds alter the distribution of cells across G1, S, and G2/M phases in a cell line–dependent manner. In MCF-7 cells, treatment with compound **4** caused a mild S-phase accumulation, whereas treatment with compound **6** increased the G1 fraction and G2/M population, suggesting a block at these checkpoints. MDA-MB-231 cells showed a marked reduction in G1 and S fractions after treatment, consistent with cell loss or sub-G1 DNA content. By contrast, normal MCF-12F cell line exhibited only minor changes, maintaining a predominantly G1-phase distribution.

The quantitative analysis of cell cycle distribution is presented in Table 2. 

### 2.6. Apoptosis

Distribution of cell populations across four quadrants based on Annexin V-FITC and Propidium Iodide (PI) staining: Q1 (Necrotic cells), Q2 (Late apoptotic-like cells), Q3 (Early apoptotic-like cells), and Q4 (Viable cells). Data represent (Table 3) the percentage (%) of total gated events for MCF-12F, MCF-7, and MDA-MB-231 cell lines under control and treatment conditions.

**Figure 7:**
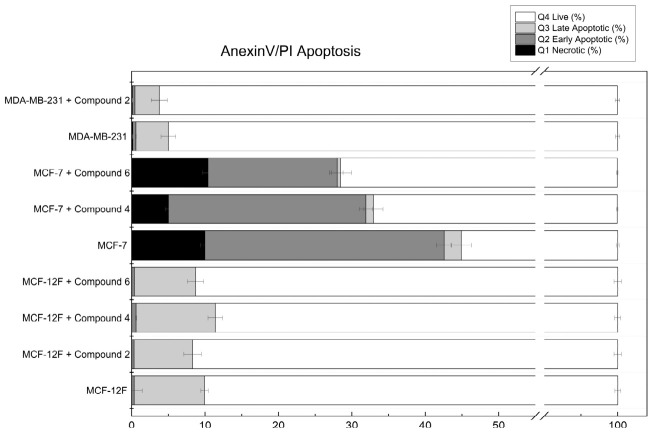
Distribution of Annexin V/PI-defined cell populations in breast cell lines.

The bar chart (Figure 7) illustrates the percentage of cells in various stages of cell death after treatment with Compounds **2**, **4**, and **6**. Cell populations were categorized using Annexin V-FITC and Propidium Iodide (PI) staining to distinguish between four distinct states: Q4: Annexin V-negative and PI-negative (white). Q3: Annexin V-positive and PI-negative (light gray). Q2: Annexin V-positive and PI-positive (dark gray). Q1: Annexin V-negative and PI-positive (black).

The MCF-7 breast cancer cell line displayed a distinct Annexin V-FITC/PI profile, with a higher proportion of Q1/Q2-positive events compared with the other investigated cell lines. The normal-like MCF-12F cells remain highly viable (>90%) across all treatments, suggesting that Compounds **2**, **4**, and **6** may possess selective cytotoxicity toward cancerous cells over healthy mammary epithelium. In the MDA-MB-231-line, Compound **2** shows minimal induction of apoptosis compared to its effects in other lines, indicating cell-line-specific responses.

The Annexin V-FITC/PI analysis revealed treatment-dependent changes in the distribution of viable, early apoptotic-like, late apoptotic-like, and necrotic cell populations across the investigated breast cell lines (Table 3, Figure 7). However, because Annexin V-FITC/PI staining alone does not establish the molecular mechanism of cell death, these findings should be interpreted as changes in cell death profiles rather than definitive evidence of apoptosis induction.

### 2.7. Actin Cytoskeleton Organization

**Figure 8:**
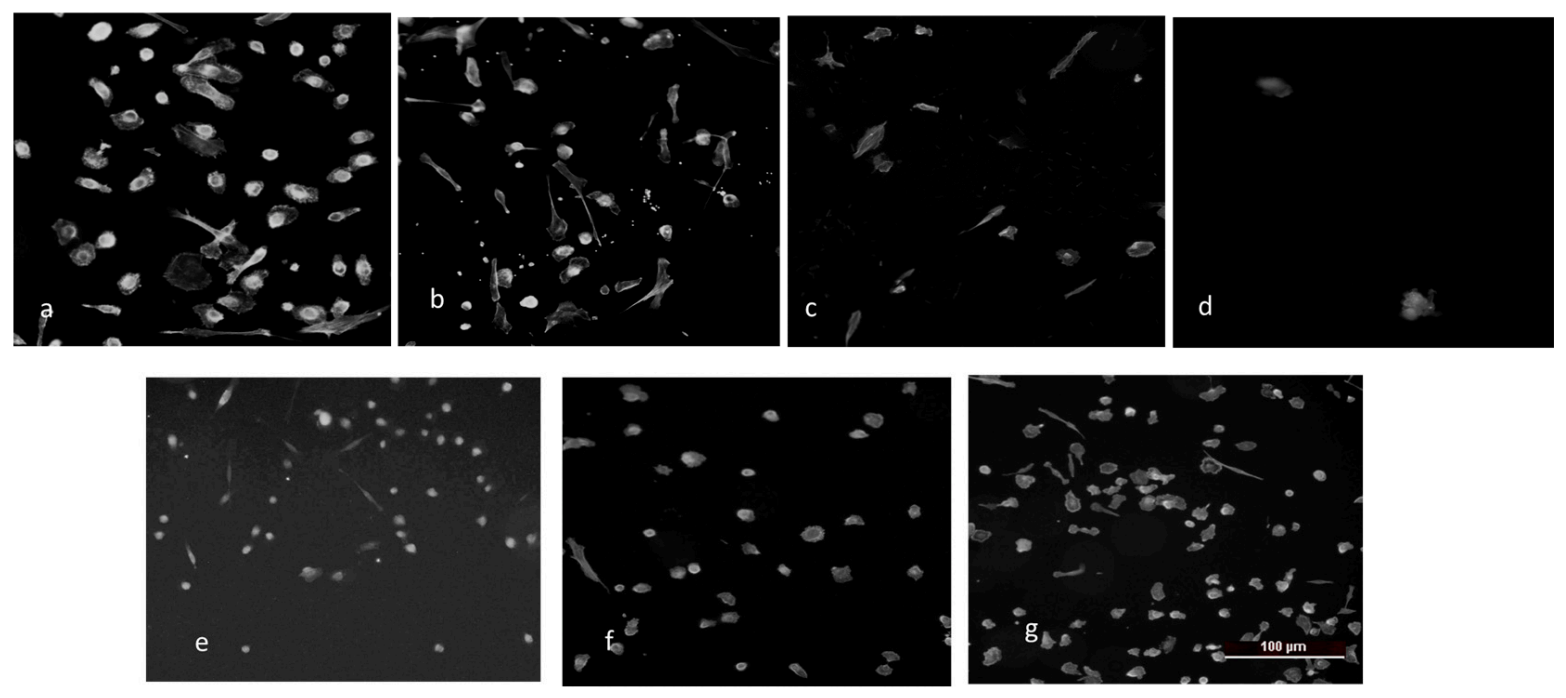
Actin staining of MCF-12 cell line after treatment with IC_50_ concentration of studied compounds: (**a**) untreated control, (**b**) Doxo—positive control, (**c**) compound **2**, (**d**) compound **4**, (**e**) compound **5**, (**f**) compound **6**, (**g**) compound **7**.

Untreated MCF-12 cells (Figure 8a, control) exhibit a typical epithelial “cobblestone-like” morphology with a well-spread shape and tight cell–cell contacts. The actin cytoskeleton is well organized, with clearly visible intracellular filaments and a distinct cortical F-actin signal, reflecting stable cell–substrate adhesion and preserved structural integrity.

Following treatment with doxorubicin (Figure 8b, positive control), pronounced cytoskeletal disorganization is observed. Cells lose their characteristic spread morphology, become rounded, and F-actin appears fragmented and predominantly peripheral, which is indicative of cellular contraction and collapse of actin architecture. Treatment with the investigated compounds (Figure 8c–g) results in alterations in cell morphology, F-actin organization, and cell density, the latter serving as an important indicator of biological effect. The general trend includes reduced cell spreading, cell rounding, weakened adhesion, and redistribution of F-actin from intracellular filaments toward a dominant peripheral (cortical) signal—features consistent with cytoskeletal stress and structural destabilization. Compound **2** shows the strongest effect, with virtually no cells observed in the examined fields, indicating a drastic reduction in cell viability and/or adhesion (Figure 8c). Compound **4** induces pronounced cell rounding and reduction in intracellular filaments, with F-actin concentrated at the periphery in the form of a thin cortical ring (Figure 8d). Cells treated with compound **5** are predominantly small and rounded, displaying a diffuse and weakened actin signal, corresponding to loss of adhesion and structural integrity (Figure 8e). Compound **6** leads to a compact cellular phenotype with a clearly expressed peripheral F-actin signal and strongly reduced intracellular filaments—indicative of a contractile, stress-associated state (Figure 8f). A mixed population of strongly rounded cells with disorganized actin signal and absence of clearly formed stress fibers is observed after treatment with compound **7**, which is consistent with severe cytoskeletal stress and possible induction of cell death (Figure 8g).

**Figure 9:**
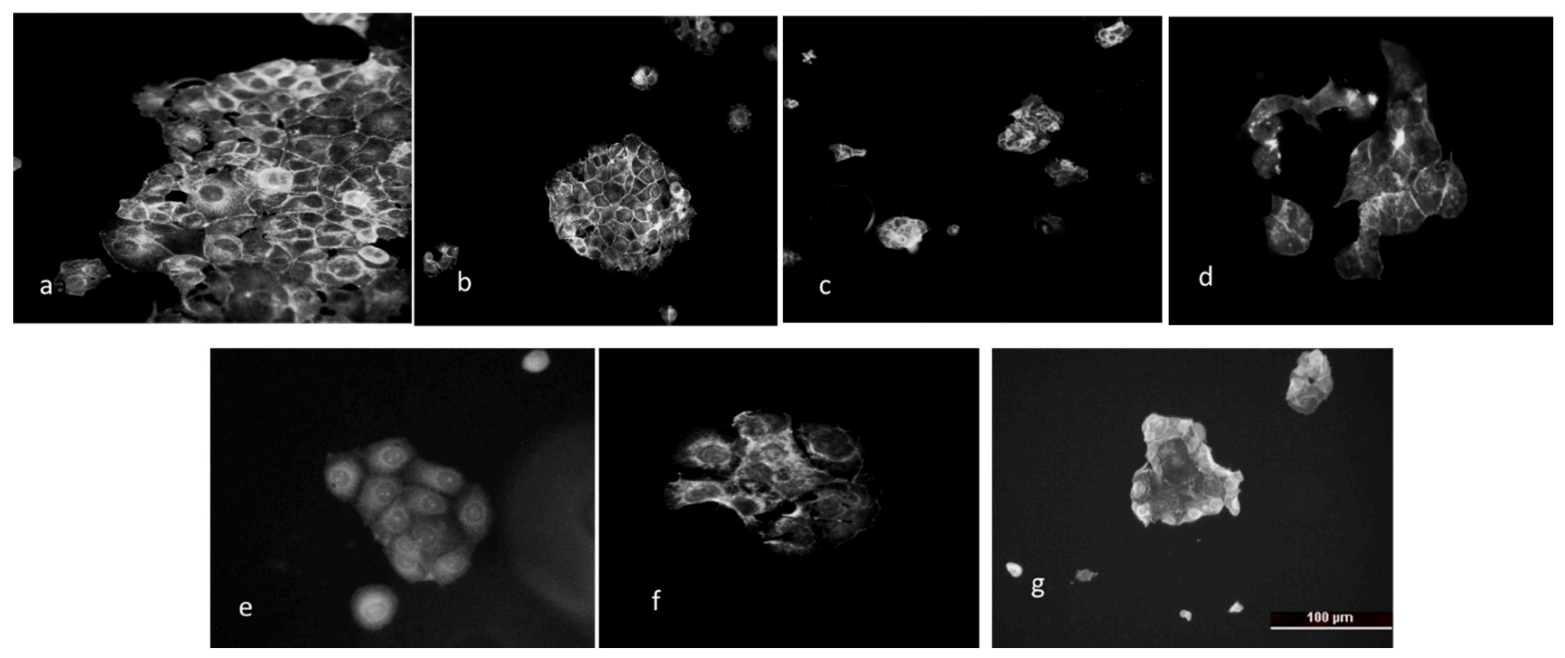
Actin staining of MCF-7 cell line after treatment with IC_50_ concentration of the studied compounds: (**a**) untreated control, (**b**) Doxo—positive control, (**c**) compound **2**, (**d**) compound **4**, (**e**) compound **5**, (**f**) compound **6**, (**g**) compound **7**.

MCF-7 cells are epithelial, hormone receptor–positive, low-invasive breast adenocarcinoma cells that typically grow in colonies with strong cell–cell contacts and relatively well-organized cortical actin. A comparative analysis is presented below:

Control cells (Figure 9a) display a typical epithelial, colony-like morphology with tightly apposed cells and well-defined cell borders. F-actin is evenly distributed along the cell periphery, with visible intracellular filamentous structures, reflecting preserved adhesion and structural stability.

Upon treatment with doxorubicin (Figure 9b), a marked disruption of colony organization is observed—cells separate from each other and adopt a more rounded morphology. Actin becomes fragmented and predominantly cortical, indicating cytoskeletal collapse and cellular contraction. Compound **2** (Figure 9c) induces a sharp reduction in cell number, with remaining isolated cells or small aggregates. F-actin is weakened and disorganized, suggesting loss of adhesion and a strong cytotoxic effect. Cells treated with compound **4** (Figure 9d), appear more dispersed and morphologically altered, some of them showing deformation. The actin signal is uneven and mainly peripheral, with poorly defined intracellular filaments, indicating cytoskeletal remodeling. Compound **5** (Figure 9e) partially preserves colony structure, but cells are smaller and more compact. F-actin appears more diffuse, with reduced filamentous organization, suggesting moderate cytoskeletal stress. In compound **6**–treated cells, a dense cellular aggregate is observed with enhanced cortical actin signal and reduced intracellular filaments, a phenotype associated with increased contractility and altered cellular dynamics (Figure 9f). Compound **7** (Figure 9g) induces pronounced cell rounding, detachment, and fragmented actin signal with absence of organized structures, consistent with severe cytoskeletal stress and possible induction of cell death.

Overall, the tested compounds disrupt the epithelial organization of MCF-7 cells, inducing rounding, weakened adhesion, and F-actin disorganization compared to the well-structured control. The strongest effects are observed with compounds **2** and **7**, characterized by reduced cell density and pronounced cytoskeletal collapse, similar in trend to the action of doxorubicin.

**Figure 10:**
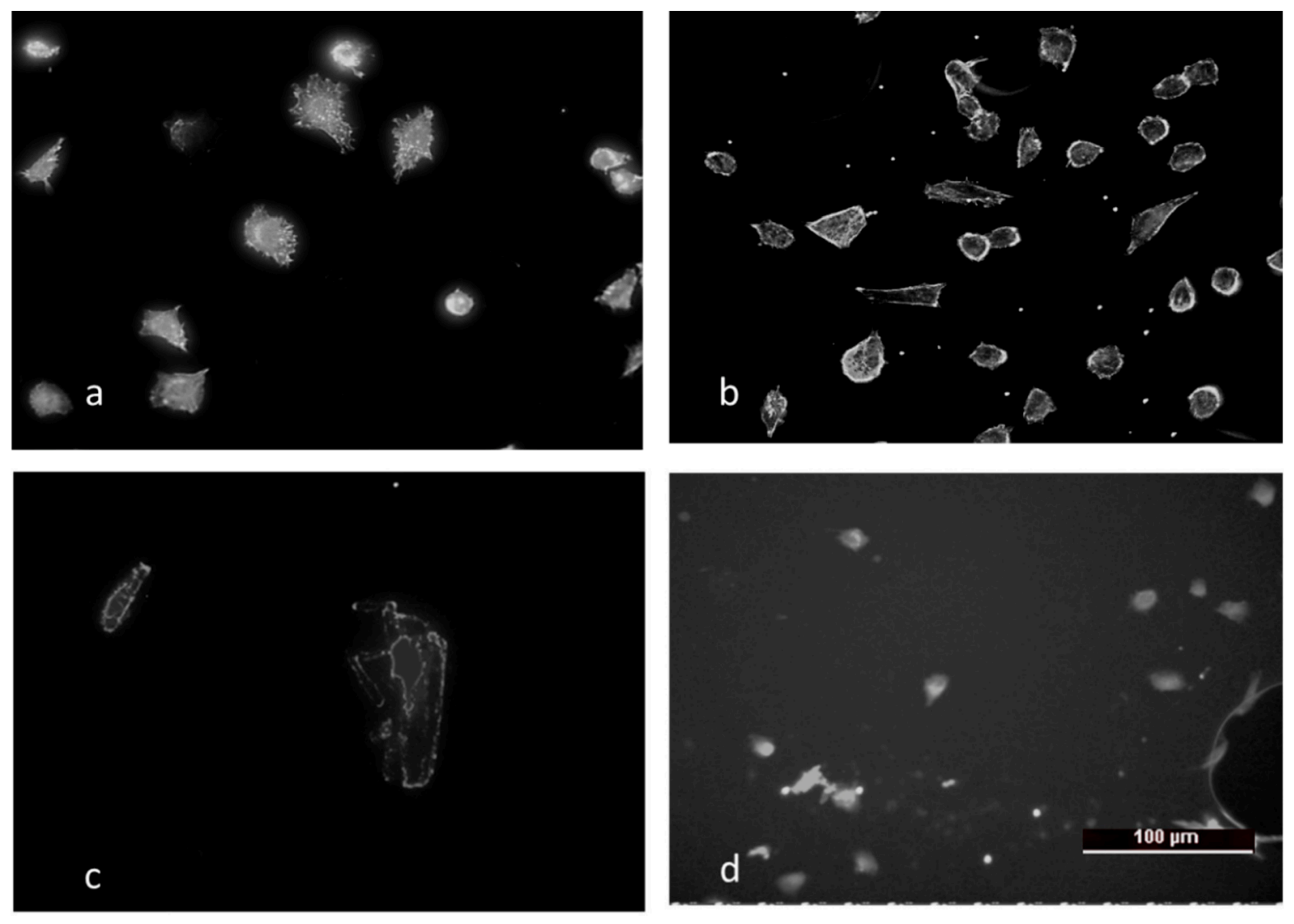
Actin staining of MDA-MB-231 cell line after treatment with IC_50_ concentration of the studied substances: (**a**) untreated control, (**b**) Doxo—positive control, (**c**) compound **2**, (**d**) compound **4**.

MDA cells (e.g., MDA-MB-231) are highly invasive, triple-negative breast carcinoma cells with a mesenchymal, fibroblast-like phenotype and high migratory capacity. They are characterized by weaker cell–cell contacts, a prominent actin cytoskeleton, and pronounced cytoskeletal dynamics associated with invasive behavior—(Figure 10a, control). The cells are well spread, with an elongated and irregular shape. A clearly defined peripheral F-actin signal and visible intracellular filamentous structures are observed, reflecting an active cytoskeleton and preserved adhesion to the substrate (Figure 10b). Cells appear smaller, and some lose their characteristic elongated morphology. The actin signal is more uneven, with less organized intracellular filaments, suggesting cytoskeletal remodeling. Marked cell rounding and reduced spreading are observed. F-actin is mainly concentrated at the periphery, while intracellular structures are strongly reduced—indicative of weakened adhesion and cytoskeletal stress (Figure 10d). Cell density is significantly decreased, with only individual small, rounded cells or cell fragments visible. The actin signal is weak and disorganized, consistent with severe cytoskeletal collapse and a pronounced cytotoxic effect.

MCF-12, MCF-7, and MDA-MB-231 cells exhibit a phenotype-dependent response to the tested substances, reflecting differences in their epithelial organization, degree of adhesion, and cytoskeletal dynamics. The highest sensitivity is observed in the invasive MDA cells, moderate in MCF-7, while the non-tumorigenic MCF-12 cells retain relatively greater structural stability, highlighting the selective action of the compounds toward the tumor phenotype.

## 3. Discussion

The present study investigated the antiproliferative and biological effects of pyrimidine-derived compounds on representative breast cell models, including the luminal A breast cancer cell line MCF-7, the highly aggressive triple-negative breast cancer cell line MDA-MB-231, and the non-tumorigenic mammary epithelial cell line MCF-12F. The obtained results demonstrate that several of the tested derivatives exhibit measurable antiproliferative activity and moderate selectivity toward malignant breast cancer cells compared with normal epithelial cells.

The initial cytotoxicity screening revealed that treatment with the investigated pyrimidine derivatives resulted in a clear concentration-dependent decrease in cell viability in both breast cancer cell lines. Among the evaluated compounds, compound **6** showed the most pronounced antiproliferative effect against MCF-7 cells, with an IC_50_ value of approximately 49 μM. Compounds **2**, **4**, and **7** demonstrated intermediate activity, with IC_50_ values ranging between 144 and 301 μM. In contrast, the MDA-MB-231 triple-negative breast cancer cells exhibited generally lower sensitivity to the tested compounds. Although a dose-dependent reduction in viability was observed, the inhibitory effects were less pronounced than those detected in MCF-7 cells. This difference in sensitivity between the two breast cancer models is consistent with the known biological characteristics of triple-negative breast cancer cells, which typically display enhanced drug resistance, increased invasiveness, and activation of multiple survival pathways that limit the efficacy of many cytotoxic agents.

Importantly, the non-tumorigenic MCF-12F epithelial cells showed significantly lower sensitivity to the tested compounds. In most cases, cell viability remained above 70–80% even at concentrations that markedly reduced the viability of cancer cells. This observation indicates a certain degree of tumor selectivity, which represents a desirable property in anticancer drug development. In comparison, the clinically used chemotherapeutic agent doxorubicin demonstrated significantly higher potency but affected both normal and cancer cells with similar efficiency, reflecting the well-known limitation of conventional chemotherapy related to poor selectivity and associated systemic toxicity.

The calculation of the selectivity index (SI) further supported the preferential activity of several compounds toward cancer cells. Among them, compound **6** demonstrated the highest selectivity toward MCF-7 cells (SI = 2.69), indicating that it was nearly three times more toxic to tumor cells than to normal epithelial cells. Compounds **4** and **7** also showed notable selectivity, with SI values of 2.14 and 1.99, respectively. In contrast, compounds **3** and **5** exhibited SI values below 1, suggesting comparable toxicity toward both normal and malignant cells. Selectivity toward MDA-MB-231 cells was generally lower, with compound **2** displaying the highest SI value (1.45), indicating only moderate preferential activity toward triple-negative breast cancer cells. Overall, these findings suggest that structural differences among the tested derivatives significantly influence their biological activity and tumor selectivity.

To further evaluate the long-term effects of the selected compounds on cellular proliferative potential, a colony formation assay was performed. This assay provides important information about the ability of cancer cells to survive treatment and retain their clonogenic capacity. The results demonstrated that treatment with the selected pyrimidine derivatives reduced clonogenic survival in a concentration-dependent manner across all investigated cell lines. In MCF-7 cells, compound **2** produced the most pronounced inhibition of colony formation, followed by compounds **6** and **4**, indicating strong suppression of long-term proliferative potential. Interestingly, in the highly metastatic MDA-MB-231 cells, compound **4** exerted the strongest inhibitory effect, whereas compound **2** reduced clonogenicity to approximately 40%. These results suggest that certain derivatives may interfere not only with short-term metabolic activity but also with the long-term reproductive capacity of cancer cells. In contrast, the non-tumorigenic MCF-12F cells retained relatively higher clonogenic potential, further supporting the selective action of the compounds toward tumor cells.

Because membrane organization and lipid dynamics play an important role in cancer cell signaling, proliferation, and migration, changes in plasma membrane lipid order were assessed using Laurdan fluorescence spectroscopy. The results revealed that treatment with the pyrimidine derivatives predominantly increased Laurdan GP values in both breast cancer cell lines, indicating altered membrane order and, for most compounds, reduced lipid fluidity. Among the tested compounds, compound **2** produced the most pronounced elevation in GP values in both MCF-7 and MDA-MB-231 cells. Increased membrane order may influence the organization of lipid rafts and membrane-associated signaling complexes, potentially affecting receptor activation and downstream signaling pathways involved in cancer cell survival and proliferation. An additional observation from the membrane lipid order analysis is the distinct effect of compound **4** on plasma membrane organization. While most of the tested pyrimidine derivatives tended to increase membrane order, compound **4** produced a measurable shift toward reduced lipid packing, indicating an increase in membrane fluidity. This effect was detected in both the low-metastatic MCF-7 cells and the highly metastatic MDA-MB-231 cells, suggesting that the interaction of this compound with the plasma membrane is not strongly dependent on the metastatic phenotype of the cells. Increased membrane disorder may influence the spatial organization of membrane lipids and associated signaling proteins, potentially affecting receptor clustering, membrane permeability, and downstream signaling pathways involved in cancer cell survival and migration. Because alterations in membrane fluidity are known to modulate the activity of numerous membrane-associated proteins, the ability of compound **4** to perturb membrane organization may represent an additional mechanism contributing to its biological activity. These findings suggest that, in addition to its antiproliferative effects, compound **4** may exert part of its action through direct modulation of plasma membrane biophysical properties in breast cancer cells. In contrast, normal MCF-12F cells exhibited only minimal changes in membrane lipid organization following treatment, suggesting that the compounds preferentially affect the membrane properties of malignant cells.

The wound-healing assay further demonstrated that the tested pyrimidine derivatives can suppress the migratory capacity of breast cancer cells. In untreated control conditions, MDA-MB-231 cells exhibited rapid wound closure, consistent with their highly invasive phenotype. Treatment with the investigated compounds significantly delayed wound closure in both breast cancer models. Compound **2** showed the strongest inhibitory effect on cell migration in both MCF-7 and MDA-MB-231 cells, indicating a potential anti-metastatic effect. Since cell migration plays a crucial role in tumor invasion and metastasis, the observed inhibition of wound closure suggests that these compounds may interfere with cytoskeletal organization and motility-related signaling pathways.

Cell-cycle analysis provided additional insight into the mechanism underlying the antiproliferative activity of the tested compounds. In MCF-7 cells, treatment with compound **4** induced a mild accumulation of cells in the S phase, suggesting interference with DNA replication. In contrast, compound **6** increased the proportion of cells in both the G1 and G2/M phases, indicating the activation of cell-cycle checkpoints and potential arrest at multiple stages of cell-cycle progression. MDA-MB-231 cells showed a marked reduction in G1 and S populations following treatment, which may reflect increased cell death or accumulation of cells with sub-G1 DNA content. Notably, normal MCF-12F cells displayed only minor changes in cell-cycle distribution, further supporting the selective impact of the tested compounds on malignant cells.

Fluorescence staining of the actin cytoskeleton revealed pronounced morphological and structural alterations in treated cells. The tested compounds induced cell rounding, reduced spreading, weakened cell–substrate adhesion, and redistribution of F-actin from intracellular filamentous structures toward a cortical localization. These changes are characteristic of cytoskeletal stress and loss of structural integrity, supporting the overall cytotoxic and antiproliferative effects of the investigated compounds. The strongest effects were observed with compounds **2** and **7**, which induced significant disruption of actin organization and a marked reduction in cell density. In highly invasive MDA-MB-231 cells, the treatment led to a collapse of cytoskeletal architecture and reduced cell spreading, suggesting inhibition of migratory and invasive behavior.

In addition, the observed alterations in membrane lipid organization may also be functionally connected with the changes detected in cell migration and cytoskeletal architecture. Plasma membrane fluidity and lipid packing play an important role in regulating the organization of membrane-associated signaling complexes that control cytoskeletal dynamics and cell motility. In the present study, treatment with several pyrimidine-derived compounds altered membrane lipid order, as indicated by changes in Laurdan GP values, while simultaneously inhibiting migratory activity in the wound-healing assay and inducing pronounced rearrangements of the actin cytoskeleton. In particular, the redistribution of F-actin toward a predominantly cortical localization and the loss of well-defined intracellular filamentous structures suggest disruption of actin polymerization and cell–substrate adhesion. Such cytoskeletal alterations are closely associated with reduced migratory capacity and impaired invasive behavior of cancer cells. It can therefore be assumed that the modulation of the biophysical properties of the plasma membrane induced by the tested compounds contributes to subsequent effects on cytoskeletal organization and cell motility. This coordinated influence on membrane structure, actin dynamics and migration may represent an important component of the anticancer activity of these pyrimidine derivatives, especially in the context of limiting metastatic potential.

The present study was not designed as a comprehensive medicinal chemistry investigation; nevertheless, comparison of the investigated thieno[2,3-*d*]pyrimidine derivatives suggests that structural modifications of the common heterocyclic scaffold substantially influence their biological activity. Despite sharing the same thieno[2,3-*d*]pyrimidine core, the compounds displayed distinct biological profiles depending on the nature of the substituents attached to the scaffold. In particular, the aromatic derivatives (Compounds **4** and **6**), the morpholine-containing derivative (Compound **5**), and the ester-containing derivative (Compound **2**) exhibited distinct biological responses across the investigated assays, indicating that the peripheral substituents modulate not only antiproliferative activity but also membrane remodeling, cell migration, clonogenic survival, and cell-cycle progression. These observations suggest that optimization of the substitution pattern and physicochemical properties may represent an effective strategy for improving the biological performance of this class of compounds. However, because only a limited number of derivatives representing different substitution patterns were investigated, these findings should be regarded as preliminary and do not allow definitive structure-activity relationships to be established.

Overall, the investigated compounds exhibited lower antiproliferative potency than doxorubicin; however, their moderate antiproliferative activity, combined with their pronounced effects on cell migration, clonogenic survival, cytoskeletal organization, and plasma membrane lipid order, identifies them as promising lead structures for further optimization. Future modification of the thieno[2,3-*d*]pyrimidine scaffold may improve both antiproliferative potency and tumor selectivity while preserving the favorable effects on membrane remodeling, cytoskeletal organization, and cell migration.

## 4. Materials and Methods

### 4.1. The Chemicals

**Figure 11:**
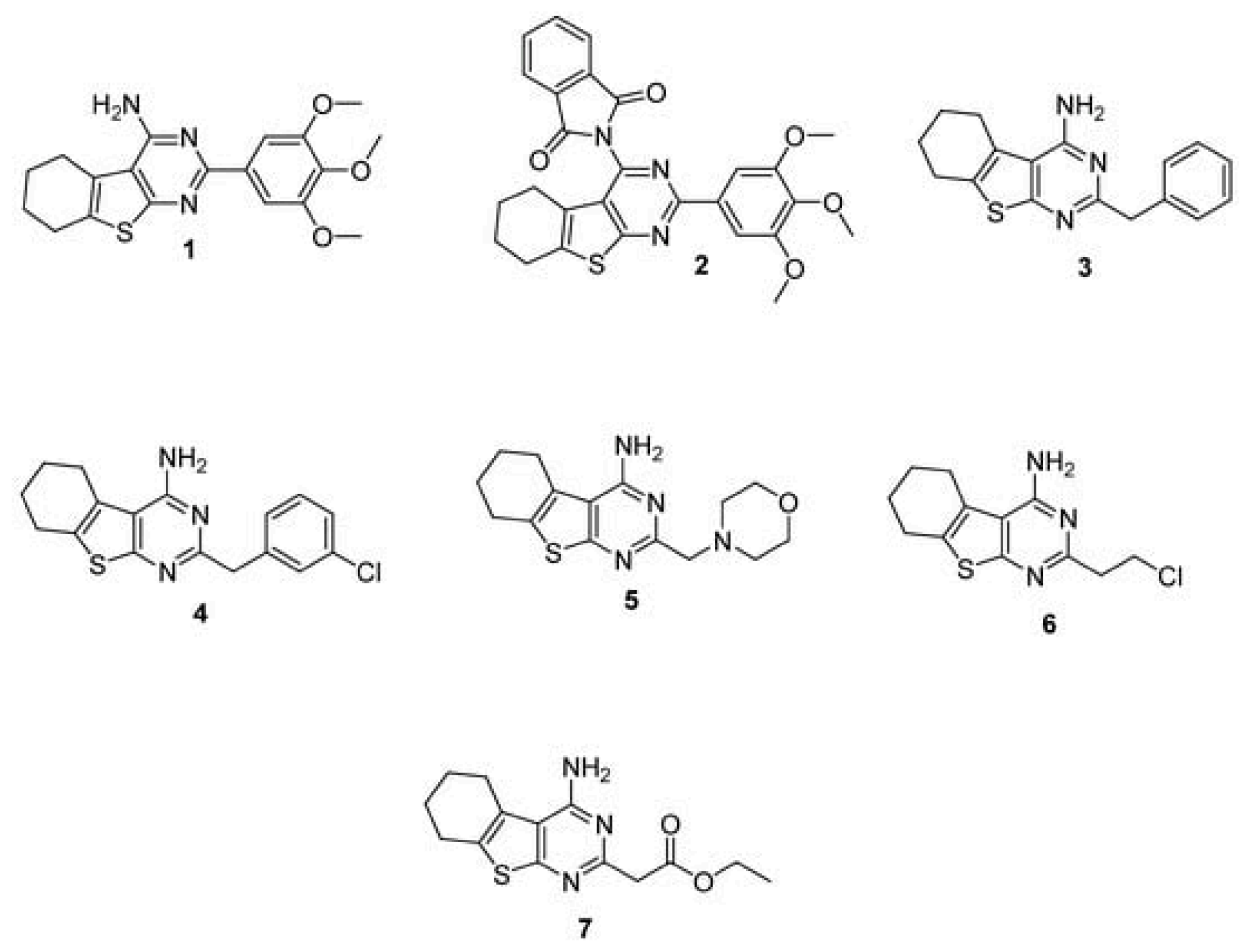
The compounds were synthesized according to previously reported procedures [37].

The reference drug (doxorubicin) was purchased from Sigma-Aldrich, Schnelldorf, Germany. Cell culture reagents were Dulbecco’s modified Eagle’s medium (DMEM), DMEM:Ham’s F12, Eagle’s minimal essential medium (EMEM), fetal bovine serum (FBS), and antibiotics (penicillin and streptomycin). Disposable consumables were supplied by Orange Scientific, Braine-l’Alouette, Belgium. Compounds: 4-Amino-2-Substituted-Tetrahydrobenzothieno[2,3-*d*]pyrimidines **1**–**7** are shown in Figure 11.

### 4.2. Cell Lines

All breast cancer cell lines (MCF-12F, MCF-7, MDA-MB-231) were purchased from American Type Culture Collection-ATCC (Manassas, VA, USA). The MCF-12F normal cell line was cultivated in DMEM:Ham’s F12 (1:1 ratio), MCF-7 and MDA-MB-were cultivated in DMEM. All media were supplemented with 10% fetal bovine serum (FBS), 1% sodium piruvate, and 1% MEM non-essential amino acids. All cell lines were maintained in a humidified atmosphere, with 5% CO_2_ at 37 °C.

### 4.3. In Vitro Anti-Proliferative Activity

The anti-proliferative activity was evaluated using the standard MTT colorimetric assay, as described by Mosmann et al. [38]. This method quantifies cell viability and metabolic activity by measuring the reduction in tetrazolium salt (MTT) into insoluble formazan crystals. Formazan absorbance was recorded at λ = 540 nm using a microplate reader. Results are reported as IC_50_ values, representing the concentration required to inhibit cell growth by 50%.

### 4.4. Colony Forming Assay

The in vitro cell survival assay was conducted following the protocol published by Franken et al. [39] with minor modifications. MCF-7 and MDA-MB-23 cells were seeded in six-well plates at a density of 500 cells/mL and allowed to adhere overnight. Following attachment, cells were treated with specified concentrations of the compounds. After a 10-day incubation period, the medium was aspirated, and colonies were fixed and stained with 2% methylene blue in 50% ethanol. Colonies containing more than 50 cells were subsequently enumerated to determine survival rates.

### 4.5. Wound Healing (WH) Assay

MCF-7 and MDA-MB-231 cells were seeded in uncoated 6-well culture plates at a density of 5 × 10^5^ cells per well and cultured until they reached a confluent monolayer (100% confluence). A linear scratch was created manually using a sterile 200 µL pipette tip, and detached cells were removed by washing the monolayer twice with phosphate-buffered saline (PBS). The culture medium was then replaced with fresh medium containing the tested compounds. Cell migration was monitored using the lens-free holographic imaging system Cytonote 6W (Iprasense, Helioparc, Pau, France). Image acquisition was initiated immediately after treatment, and images were recorded every 10 min for 24 h. The system provides a large field of view (29.4 mm^2^), enabling continuous monitoring of wound closure. The acquired images were analyzed using HORUS software (version 6.22.2, Iprasense) and OriginPro software 9.0 to determine wound closure kinetics and migration rates, and representative images at selected time points were generated.

### 4.6. Cell Cycle Analysis by Propidium Iodide (PI) Staining

Cells were harvested by trypsinization, resuspended in medium supplemented with 10% FCS, and collected via centrifugation (1000 rpm for 5 min). The resulting pellet was washed in 1 mL of PBS before fixation in 2.5 mL of pre-chilled 70% ethanol for 15 min on ice. For the staining procedure, cells were resuspended in 500 µL of PI solution containing 0.1 mg/mL RNase A and 0.05% Triton X-100 both purchased from Sigma-Aldrich, St. Louis, MO, USA. Following a 10 min incubation at 37 °C, cell fluorescence was measured using a flow cytometer (LSR II, BD Biosciences, Franklin Lakes, NJ, USA). Data were acquired and processed using BD FACS DIVA software v. 6.1.3 (BD Biosciences).

### 4.7. Actin Staining

Following a 24 h treatment with the test compounds, cells were washed with phosphate-buffered saline (PBS, purchased from Thermo Fisher Scientific, Waltham, MA, USA) and fixed in 3% paraformaldehyde (PFA; Electron Microscopy Sciences Hatfield, PA, USA) for 15 min at room temperature. The fixed samples were permeabilized with 0.5% Triton X-100 (Merck, Darmstadt, Germany) for 5 min, followed by a 10 min incubation in 1% bovine serum albumin (BSA; Sigma-Aldrich, St. Louis, MO, USA) to block non-specific binding sites. F-actin filaments were then stained by incubating the cells with Phalloidin–TRITC (1:200 in PBS; Sigma-Aldrich, St. Louis, MO, USA) for 30 min at room temperature in the dark. After three washes with PBS and a final rinse in distilled water, coverslips were mounted onto glass slides using Mowiol^®^ 4-88 (Sigma-Aldrich, St. Louis, MO, USA). Fluorescence images were acquired using a Leica DMI 3000 B microscope (Leica Microsystems CMS GmbH, Wetzlar, Germany).

### 4.8. Membrane Lipid Order Assessment

Membrane lipid order was evaluated using the fluorescent probe Laurdan (6-dodecanoyl-2-dimethylaminonaphthalene; Thermo Fisher Scientific Waltham, MA, USA). Breast cancer cells were seeded in 96-well plates 1 × 10^4^ cells/mL) and treated with either DMSO (vehicle control) or compounds at their respective IC_50_ concentrations for 24 h. Following treatment, cells were washed with PBS and incubated with 3.5 µM Laurdan in medium for 60 min at 37 °C in the dark. After removing unincorporated dye, fluorescence was recorded using a TECAN Infinite M200 Pro microplate reader (Tecan Group Ltd., Männedorf, Switzerland; excitation: 355 nm).

Emission intensities were measured at 440 nm (I440, representing the ordered phase) and 490 nm (I490, representing the disordered phase). The Generalized Polarization (GP), a measure of membrane fluidity, was calculated according to the following equation:

GP = (I440 − I490)/(I440 + I490)

GP values represent the mean of at least three independent biological replicates, with 20 technical measurements per replicate. Data analysis was conducted using Origin (v9.0), and statistical significance was determined via the Mann–Whitney test.

### 4.9. Statistical Analysis

All experiments were performed in three independent biological replicates, each containing triplicate technical measurements, unless otherwise specified. Data obtained from flow cytometry (FACS) and colony forming analyses are presented as the mean ± standard deviation (SD). Statistical analysis of the MTT assay was performed using one-way analysis of variance (ANOVA) followed by Dunnett’s post hoc test. A *p* value < 0.05 was considered statistically significant. Statistical significance is indicated as follows: *ns*, not significant (*p* > 0.05); * *p* < 0.05; ** *p* < 0.01; *** *p* < 0.001. Detailed statistical analyses are provided in the Supplementary Materials.

## 5. Conclusions

Overall, the present study demonstrates that the investigated thieno[2,3-*d*]pyrimidine derivatives exert multimodal biological activity in breast cancer cell models by inhibiting cell proliferation and clonogenic survival, modulating plasma membrane organization, reducing cell migration, altering cytoskeletal architecture, and affecting cell-cycle progression. These findings indicate that the biological activity of this class of compounds extends beyond their antiproliferative effects and involves coordinated modulation of several cellular processes associated with tumor progression and metastatic potential.

Among the investigated derivatives, Compound **6** exhibited the most favorable overall biological profile, combining antiproliferative activity with moderate tumor selectivity and pronounced effects on clonogenic survival and cell-cycle progression. In contrast, Compound **2** showed the strongest effects on plasma membrane lipid organization and inhibition of cell migration, suggesting that membrane remodeling may contribute to its biological activity. These differences further support the influence of structural modifications on the biological properties of thieno[2,3-*d*]pyrimidine derivatives.

Although the investigated compounds displayed lower antiproliferative potency than doxorubicin, their broad spectrum of biological effects identifies the thieno[2,3-*d*]pyrimidine scaffold as a promising platform for further structural optimization toward the development of novel anticancer agents with improved potency, tumor selectivity, and anti-metastatic activity.

## Figures and Tables

**Table 1:** Selectivity index (SI) of compounds **1**–**7** and Doxorubicin.

Compounds	IC_50_ ± SD (μM)			SI	
	MCF-12F	MCF-7	MDA-MB-231	MCF-7	MDA-MB-231
**1**	109.18 ± 10.46	71.56 ± 5.85	126.62 ± 7.73	1.53	0.86
**2**	278.53 ± 17.96	144.3 ± 3.81	192.09 ± 9.70	1.93	1.45
**3**	997.21 ± 70.72	1103.21 ± 60.87	2995.40 ± 72.95	0.9	0.33
**4**	1091.33 ± 94.64	510.21 ± 41.68	1470.49 ± 117.01	2.14	0.74
**5**	98.05 ± 5.19	105.61 ± 10.85	99.29 ± 3.02	0.93	0.99
**6**	131.91 ± 8.86	48.98 ± 3.65	1085.58 ± 69.27	2.69	0.12
**7**	600.44 ± 35.21	301.84 ± 33.94	1090.45 ± 43.92	1.99	0.55
**Doxo**	0.59 ± 0.06	0.67 ± 0.03	2.54 ± 0.11	0.89	0.23

**Table 2:** Quantitative analysis of the cell cycle distribution for the three breast cell lines (MCF-12F, MCF-7, and MDA-MB-231) following treatment with compounds (Compounds **2**, **4**, and **6**).

Cell Line	Treatment	Phases		
		% G1	% S	% G2
MCF-12F	Untreated	78.30	8.57	11.00
MCF-12F	Compound **2**	65.60	22.50	9.53
MCF-12F	Compound **4**	74.10	10.70	11.30
MCF-12F	Compound **6**	76.90	9.40	10.10
MCF-7	Untreated	49.40	38.20	8.97
MCF-7	Compound **4**	43.60	44.70	8.73
MCF-7	Compound **6**	60.80	26.50	12.60
MDA-MB-231	Untreated	50.10	42.90	4.63
MDA-MB-231	Compound **2**	5.43	18.60	0

**Table 3:** Flow cytometric quantification of Annexin V/PI-defined apoptotic and necrotic cell populations in breast cell lines.

Cell Line	Treatment	Q1 ANX-V (−)/PI (+)	Q2 ANX-V (+)/PI (+)	Q3 ANX-V (+)/PI (−)	Q4 ANX-V (−)/PI (−)
		Necrotic (%)	Late Apoptotic (%)	Early Apoptotic (%)	Live (%)
MCF-12F	Untreated	0.02	0.32	9.58	90.10
MCF-12F	Compound **2**	0.03	0.30	7.98	91.70
MCF-12F	Compound **4**	0.01	0.59	10.80	88.60
MCF-12F	Compound **6**	0.03	0.34	8.34	91.30
MCF-7	Untreated	9.97	32.60	2.34	55.10
MCF-7	Compound **4**	5.01	26.90	1.03	67.00
MC-7	Compound **6**	10.40	17.06	0.45	72.09
MDA-MB-231	Untreated	0.15	0.43	4.42	95.0
MDA-MB-231	Compound **2**	0.10	0.35	3.32	96.2

## Data Availability

The data are stored by the authors and, if there is a valid reason, they can be sent via personal correspondence.

## Supplementary Materials

The following supporting information can be downloaded at: https://www.mdpi.com/article/10.3390/molecules31142435/s1, Figure S1. Statistical analysis of the dose-dependent antiproliferative activity of compounds 1–7 and doxorubicin in MCF-12F, MCF-7, and MDA-MB-231 cells.
