# Supplementary material for: Selected Thieno[2,3-d] Pyrimidine Derivatives Target Breast Cancer Cell Proliferation and Membrane Organization
Source: Molecules. 2026 Jul 11;31(14):2435. doi: 10.3390/molecules31142435 (PMC13414003; doi:10.3390/molecules31142435)
Supplement: Supplementary file 1 [file molecules-31-02435-s001.zip › molecules-4374303-supplementary.pdf]

Supplementary Materials

Selected Thieno[2,3-d] Pyrimidine Derivatives Target Breast Cancer Cell Proliferation and Membrane Organization

Aleksandrina Nesheva[1], Ivan Iliev[2], Anelia Mavrova[3], Denitsa Yancheva[4], Aneliya Kostadinova[1], Severina Semkova[1], Albena Momchilova[1], Iana Tsoneva[1], Biliana Nikolova[1*], Galya Staneva[1*]

[1] Institute of Biophysics and Biomedical Engineering, Bulgarian Academy of Sciences, Acad. G. Bonchev Str., Bl. 21, 1113 Sofia, Bulgaria, nescheva@gmail.com (A.N.); aneliakk@yahoo.com (A.K.); seveina.yordanova@gmail.com (S.S.); albena_momchilova@abv.bg (A.M.); itsoneva@bio21.bas.bg (I.T.); nikolova@bio21.bas.bg (B.N.); gstaneva@bio21.bas.bg (G.S.)

[2] Institute of Experimental Morphology, Pathology and Anthropology with Museum, Bulgarian Academy of Sciences, Acad. G. Bonchev Str., Bl. 25, 1113 Sofia, Bulgaria, taparsky@abv.bg

[3] Department of Organic Chemistry, Faculty of Chemical Technologies, University of Chemical Technology and Metallurgy, S8 Kliment Ohridski Blvd., 1756 Sofia, Bulgaria, anmav@abv.bg

[4] Institute of Organic Chemistry with Centre of Phytochemistry, Bulgarian Academy of Sciences, Acad. G. Bonchev Str., Bl. 9, 1113 Sofia, Bulgaria, denitsa.pantaleeva@orgchm.bas.bg

* Correspondence: nikolova@bio21.bas.bg (B.N.); gstaneva@bio21.bas.bg (G.S.)

Contents:

1. **Figure S1. Statistical analysis of the dose-dependent antiproliferative activity of compounds 1–7 and doxorubicin in MCF-12F, MCF-7, and MDA-MB-231 cells....p.2**

Figure S1. Statistical analysis of the dose-dependent antiproliferative activity of compounds 1–7 and doxorubicin in MCF-12F, MCF-7, and MDA-MB-231 cells. Cells were treated with increasing concentrations of compounds 1–7 or doxorubicin, and antiproliferative activity was evaluated after 72 h. The statistical analysis involved One-way ANOVA followed by Dunnett's post hoc test, $p < 0.05$ was accepted as the lowest level of statistical significance. ns (no significant) $p > 0.05$, *$p < 0.05$, **$p < 0.01$, ***$p < 0.001$

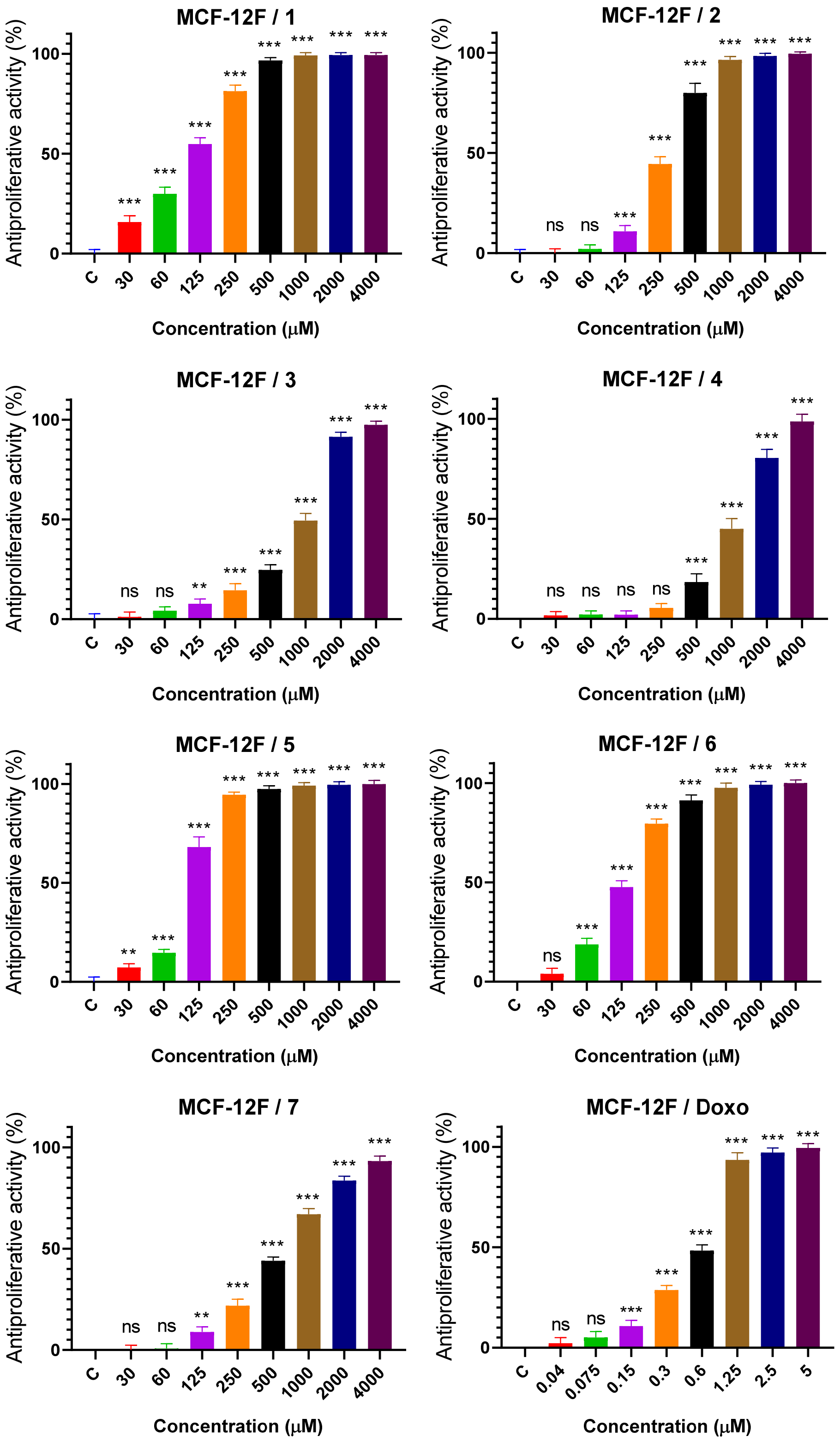

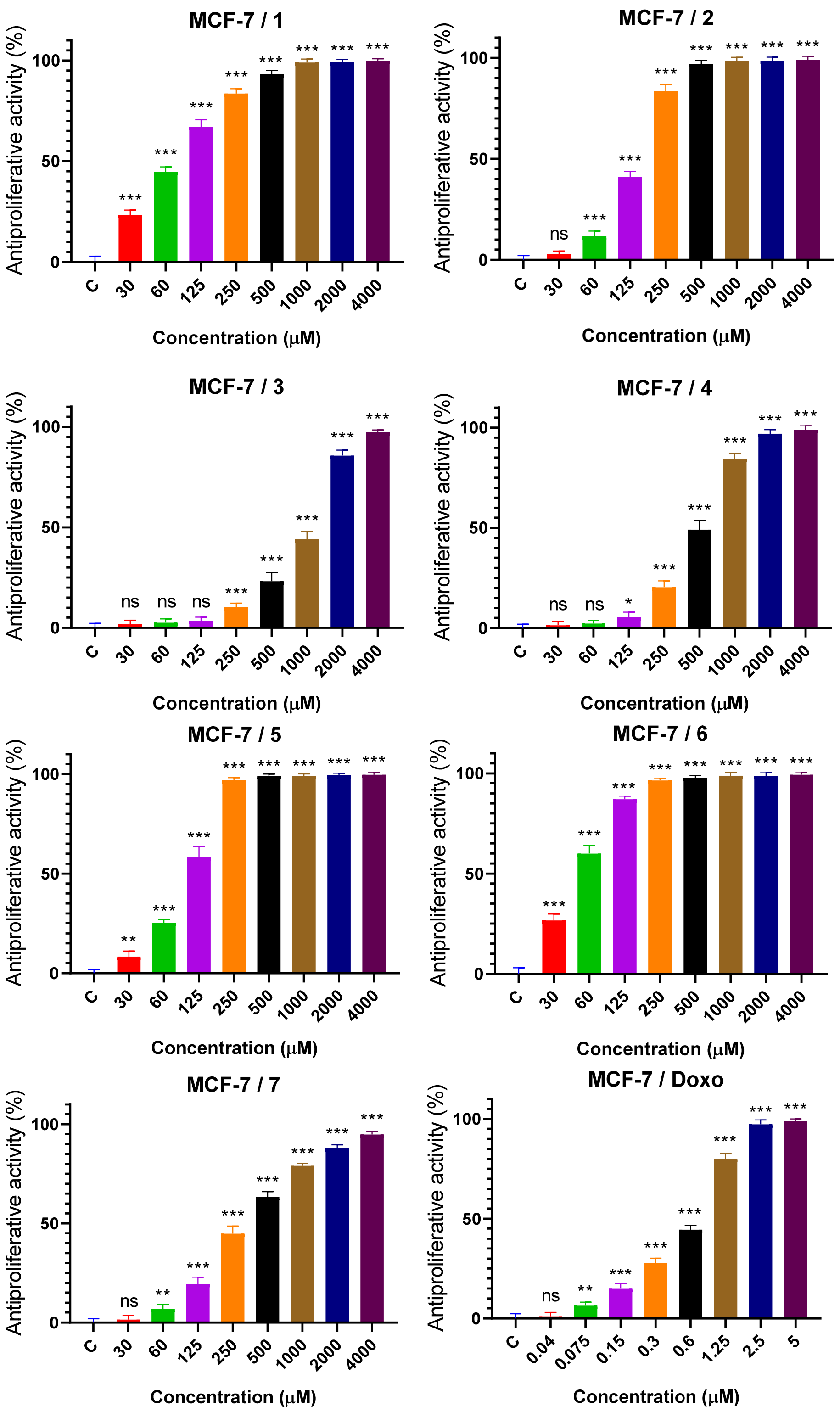
MCF-7 / 1
MCF-7 / 2
MCF-7 / 3
MCF-7 / 4
MCF-7 / 5
MCF-7 / 6
MCF-7 / 7
MCF-7 / Doxo
Antiproliferative activity (%)
0
50
100
C
30
60
125
250
500
1000
2000
4000
0.04
0.075
0.15
0.3
0.6
1.25
2.5
5
Concentration (μM)
ns
*
**
***

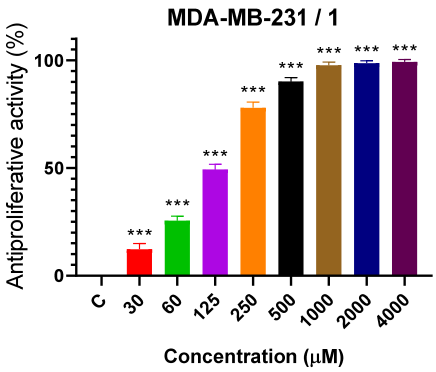
MDA-MB-231 / 1
Antiproliferative activity (%)
0
50
100
*** *** *** *** *** *** *** ***
C
30
60
125
250
500
1000
2000
4000
Concentration (μM)

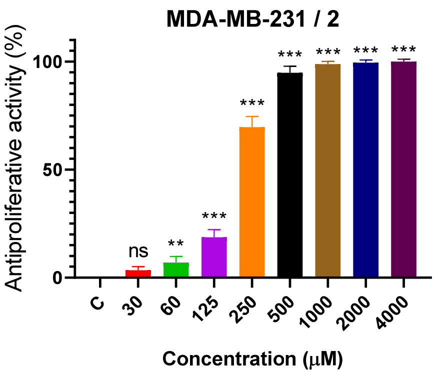
MDA-MB-231 / 2
Antiproliferative activity (%)
0
50
100
ns ** *** *** *** *** *** ***
C
30
60
125
250
500
1000
2000
4000
Concentration (μM)

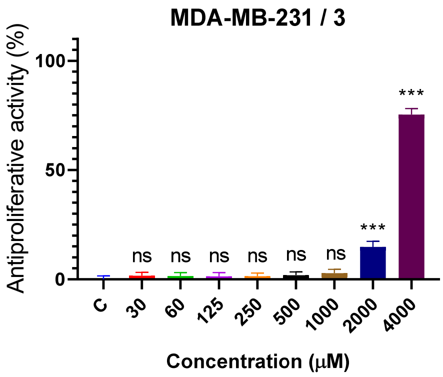
MDA-MB-231 / 3
Antiproliferative activity (%)
0
50
100
ns ns ns ns ns ns *** ***
C
30
60
125
250
500
1000
2000
4000
Concentration (μM)

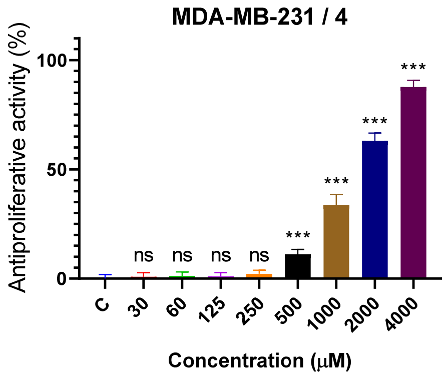
MDA-MB-231 / 4
Antiproliferative activity (%)
0
50
100
ns ns ns ns *** *** *** ***
C
30
60
125
250
500
1000
2000
4000
Concentration (μM)

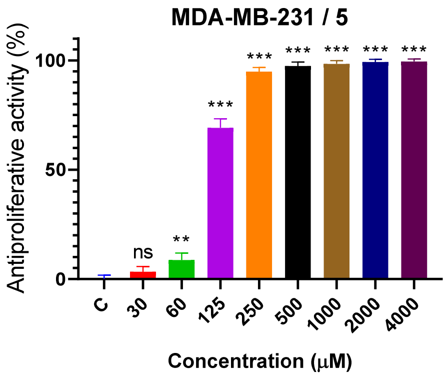
MDA-MB-231 / 5
Antiproliferative activity (%)
0
50
100
ns ** *** *** *** *** *** ***
C
30
60
125
250
500
1000
2000
4000
Concentration (μM)

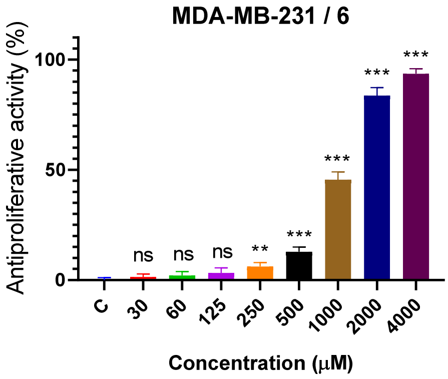
MDA-MB-231 / 6
Antiproliferative activity (%)
0
50
100
ns ns ns ** *** *** *** ***
C
30
60
125
250
500
1000
2000
4000
Concentration (μM)

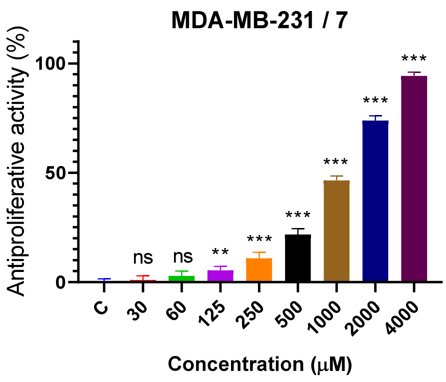
MDA-MB-231 / 7
Antiproliferative activity (%)
0
50
100
ns ns ** *** *** *** *** ***
C
30
60
125
250
500
1000
2000
4000
Concentration (μM)

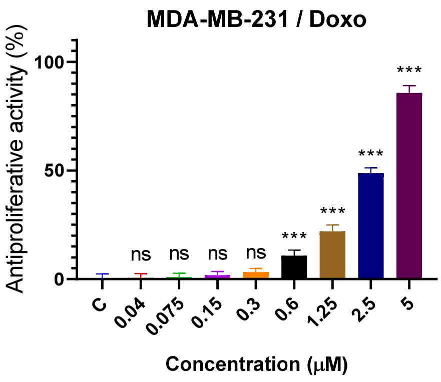
MDA-MB-231 / Doxo
Antiproliferative activity (%)
0
50
100
ns ns ns ns *** *** *** ***
C
0.04
0.075
0.15
0.3
0.6
1.25
2.5
5
Concentration (μM)
